# Advances and Challenges in Studying Hepatitis B Virus *In Vitro*

**DOI:** 10.3390/v8010021

**Published:** 2016-01-14

**Authors:** Dvora Witt-Kehati, Maya Bitton Alaluf, Amir Shlomai

**Affiliations:** 1 The Liver Institute, Rabin Medical Center Beilinson Hospital, Petah-Tikva, Israel; 2 Sackler Faculty of Medicine, Tel-Aviv University, Tel-Aviv, Israel; dvori.witt@gmail.com; 3 Department of Medicine D, Rabin Medical Center Beilinson Hospital, Petah-Tikva, Israel; mayabitton20@yahoo.com

**Keywords:** hepatocye-like cells, virus-host interactions, primary human hepatocytes

## Abstract

Hepatitis B virus (HBV) is a small DNA virus that infects the liver. Current anti-HBV drugs efficiently suppress viral replication but do not eradicate the virus due to the persistence of its episomal DNA. Efforts to develop reliable *in vitro* systems to model HBV infection, an imperative tool for studying HBV biology and its interactions with the host, have been hampered by major limitations at the level of the virus, the host and infection readouts. This review summarizes major milestones in the development of *in vitro* systems to study HBV. Recent advances in our understanding of HBV biology, such as the discovery of the bile-acid pump sodium-taurocholate cotransporting polypeptide (NTCP) as a receptor for HBV, enabled the establishment of NTCP expressing hepatoma cell lines permissive for HBV infection. Furthermore, advanced tissue engineering techniques facilitate now the establishment of HBV infection systems based on primary human hepatocytes that maintain their phenotype and permissiveness for infection over time. The ability to differentiate inducible pluripotent stem cells into hepatocyte-like cells opens the door for studying HBV in a more isogenic background, as well. Thus, the recent advances in *in vitro* models for HBV infection holds promise for a better understanding of virus-host interactions and for future development of more definitive anti-viral drugs.

## 1. Introduction

Hepatitis B virus (HBV) is a small DNA virus that infects the liver and is a major cause for end stage liver disease and liver cancer [1]. The small viral genome is 3.2 kb in length with an overlapping gene organization. Following binding to its receptor, the bile-acid pump sodium-taurocholate cotransporting polypeptide NTCP [2,3], HBV enters into the cell and establishes a nuclear pool of episomal DNA in the form of covalently closed circular DNA (cccDNA). The cccDNA molecules reside in the infected cells’ nuclei and serve as the template for viral transcription. HBV is dependent for its replication on viral-encoded polymerase that reverse transcribes the pre-genomic RNA to form the partially double-stranded DNA in the mature virion [4]. Nucleot(s)ide analogues, currently the standard of care for chronically infected patients, effectively block the viral polymerase activity and hence viral replication. However, those drugs do not affect the cccDNA pool and, therefore, complete viral eradication resulting in chronically infected patients’ cure is still a major challenge [5,6].

Since its discovery in the late 1960s and throughout the years, HBV research has been hampered by the lack of robust and reproducible cell culture systems that reliably mimic the viral life cycle [7].

This review summarizes major milestones in the development of cell culture systems for HBV, focusing on recent technological and methodological advances enabling the development of more robust and physiologically relevant infection systems based on immortalized cell lines as well as on primary human hepatocytes.

## 2. *In vitro* Systems Based on Primary Non-Human Hepatocytes

To study the HBV life cycle and its interactions with the host *in vitro*, one should ideally incorporate a constant and authentic viral pool, a genuine host cell, and a reliable and relatively easy to perform readout(s) for viral infection. Yet, none of these seem to be easy to achieve in the case of HBV. Obviously, primary human hepatocytes are considered as the gold standard host cells for HBV infection. However, those cells are phenotypically unstable *in vitro*, losing their permissiveness for HBV infection soon after isolation and plating on culture dishes [8,9]. Earlier attempts to infect primary human hepatocytes with infectious inoculums of HBV were encountered with large variability among hepatocyte donors as well as low rates and short durability of infection, even upon supplementation of dimethyl sulfoxide (DMSO) to support the differentiation state of the cells [10,11].

The woodchuck hepatitis virus (WHV) was the first of the mammalian and avian hepadnaviruses described following the discovery of HBV [12]. Primary cultures of woodchuck hepatocytes proved to be susceptible to infection with WHV, resulting in cccDNA formation and active viral replication and were therefore used as a platform to study the effect of anti-viral drugs on cccDNA persistence [13,14]. However, only few *in vitro* studies using the WHV system have been published, most probably due to difficulties in reproducing conditions to achieve productive infection. Nevertheless, the major utility of the WHV system remained in the context of *in vivo* studies on infected animals. These were pivotal for anti-viral drug studies [15] as well as for elucidating molecular pathways in HBV-associated carcinogenesis [16,17] and the interactions between the virus and the anti-viral immune response [18,19].

As opposed to both primary human hepatocytes and WHV hepatocytes, primary duck hepatocytes infected with duck hepatitis B virus (DHBV) have been found to be much easier to handle and very useful for studying basic questions in viral life cycle and especially in cccDNA formation and amplification [20,21]. However, despite being a member of the *Hepadnaviridae* family and sharing a similar life cycle to human HBV, DHBV still differs from HBV in several properties, including its shorter genome and the absence of the functional HBV X (HBx) protein [22]. Therefore, conclusions derived from DHBV system regarding cccDNA amplification and maintenance [23] as well as viral entry [24] might not necessarily hold true for HBV and are, therefore, clinically irrelevant. This emphasizes the need for using a system incorporating authentic HBV for studying the virus and its interactions with the host.

*Tupaia Belangeri* (treeshrew), on the other hand, is the only species susceptible for HBV infection besides humans and chimpanzees. Primary Tupaia hepatocytes have been shown to support HBV infection *in vitro*, although the magnitudes of infection efficiency and viral spread in this system are not entirely clear [25]. Importantly, primary Tupaia hepatocytes have been used as the target cells for photo-cross-linking experiments with a synthetic pre-S1 peptide that were key in identification of NTCP as the receptor for HBV and hepatitis D virus (HDV) [3], a major milestone in HBV research in recent years (for further discussion see Section 3.4.).

## 3. *In vitro* Systems Based on Hepatoma Cell Lines

### 3.1. Stably HBV-Transfected Cell Lines

Immortalized hepatoma cell lines, such as HepG2 and Huh7 cells, are very convenient to work with but are normally not permissive for HBV infection. To circumvent this problem, Sells and colleagues transfected hepatoma cells with a cloned head to tail HBV dimer, resulting in viral gene expression and replication as well as the formation of infectious viral particles that can readily infect naïve chimpanzees [26,27]. The so-called HBV-expressing HepG2.2.15 clone has been extensively used since then for studying basic questions in HBV biology as well as a platform for testinganti-viral drugs [28,29]. This system, as well other similar systems based on stably integrated HBV DNA [30], have the obvious advantage of stably expressing viral gene products and maintaining continuous HBV replication, and are therefore also used as a source for tissue culture derived virions for infection experiments. However, unlike the situation *in vivo*, viral production is mainly derived from the integrated rather than from the episomal DNA, which is hard to detect by conventional methods in this cell line. The introduction of hepatoma cells stably expressing HBV from a Tet-on/Tet-off system, the HepAD38 cell line, not only allowed for a better and more tightly controlled system to study HBV, but also resulted in a more robust production of virions and enhanced cccDNA accumulation in the cells [31,32]. More recently, a newer version of HepG2 cells stably transfected with a Tet-inducible HBV genome has been introduced, designated HepDE19 cell line. In this system, the 1.1 over-length HBV transgene is mutated in its 5′ pre-core ATG, whereas the 3′ pre-core ATG remains intact. As a result, the HBV e-Antigen (HBeAg) is expressed from the episomal DNA (cccDNA) but not from the integrated genome. By analyzing secreted HBeAg as a surrogate marker for cccDNA abundance, this system has been used as a platform for a large-scale screening for cccDNA-targeting drugs [33,34].

### 3.2. Delivery Vector Systems of the HBV Genome

Although the aforementioned cell lines are based on functional, integrated HBV genome, HBV integration is not obligatory for the HBV life cycle and does not produce infectious viruses *in vivo*. Therefore, with the entry machinery of HBV into the cells still remaining much of a black box, efforts have been made over the years to find alternative ways to deliver the HBV genome to the cells in a more physiological manner. The development of a recombinant HBV baculovirus system, produced in insect cells, enabled the delivery of a functional HBV genome into hepatoma cells resulting in productive HBV replication, formation of infectious viruses and establishment of a detectable intracellular cccDNA pool [35,36,37]. This system has been used for a variety of *in vitro* studies, such as testing the efficiency of novel anti-HBV drugs [38] as well as for drug resistance studies [39]. Another potential delivery system for the HBV genome is the adenovirus vector [40]. Adenovirus vector carrying the HBV genome (Ad-HBV) has been shown to infect a wide range of hepatocytes irrespective of species barrier, resulting in episomal DNA formation and robust HBV replication [41,42]. The delivery of HBV genome using a lentiviral vector has been experimentally used for *in vitro* experiments, as well [43]. However, albeit having several advantages over the traditional HepG2.2.15 cell line and its derivatives, those delivery vector systems still suffered from significant limitations; first, delivery of the HBV genome by a viral vector completely bypassed the natural entry stage of HBV, thereby eluding studies regarding this crucial step in HBV life cycle. Second, a part of the host response to HBV infection could have been largely masked by the non-specific response to the viral vector used for HBV delivery, making it hard to interpret data regarding the innate immune response to HBV infection [44], for example. Third, safety issues especially regarding work with HBV harboring lenti-viral vectors are of a major concern and are therefore a major obstacle for a wide usage of this delivery system.

### 3.3. Differentiated Hepatoma Cell Lines

Given their easy handling, low cost and reproducibility, ongoing efforts have been made to achieve authentic infection in the traditionally non-permissive hepatoma cell lines, by their further differentiation into cells better resembling primary human hepatocytes. In one report, Shaul’s group was able to show that upon supplementation of DMSO (and even more than that, the combination of DMSO and 5-aza-2′-deoxycytidine), HepG2 cells become permissive to HBV infection [45]. A big leap forward was the introduction of a novel hepatoma cell line, designated HepaRG, that presents morphological and functional features similar to primary hepatocytes and that is susceptible to HBV infection upon supplementation of corticoids and DMSO to maintain the cells’ differentiationstate [46]. The ability of this system to recapitulate the whole life cycle of HBV in the context of authentic infection established its role as an experimental platform for studies addressing key questions in HBV biology such as the role of the innate immune response in counteracting HBV infection [44,47,48], cccDNA regulation [23,49], and mechanisms of viral entry [50,51]. However, this infection system still suffered from substantial limitations, such as the need for polyethylene glycol (PEG) supplementation to achieve infection, relatively low infection efficiency, and stringent conditions to maintain those cells’ state of differentiation.

### 3.4. NTCP Expressing Hepatoma Cell Lines

It was not until a decade later that the bile acid pump NTCP has been shown to serve as a receptor for both HBV and HDV [2,3]. This revolutionizing discovery and the realization that NTCP expression on the plasma membrane of hepatoma cells is much less abundant as compared to primary hepatocytes, has opened the door to establishing HepG2 and Huh7-based cell lines in which NTCP is over expressed and that can be readily infected with HBV [52]. More recently, a novel system based on NTCP expressing hepatoma cells co-cultured with HBV-specific CD8 cells has been suggested as a platform for studying the immunobiology of HBV in the context of a tissue-culture format, as well [53]. However, although improved techniques, such as spinoculation during HBV inoculation, greatly enhanced infection efficiency of NTCP expressing cells [54], the system still has its limitations; first, the multiplicity of infection (MOI) needed to achieve substantial infection is extremely high (in the range of hundreds, and even thousands) and, in most instances, PEG is needed to enhance infection. Second, in contrast to the situation *in vivo*, infection is short-lived, it does not result in substantial viral spreading and the amount of cccDNA detected is modest. This suggests that other factors essential for productive HBV infection are probably impaired or even missing in those cancerous cell lines. Last but not least, despite their flexibility and easy handling, hepatoma cells are physiologically impaired in many intracellular pathways and functions, limiting their use as a platform for studying virus-host interactions. Therefore, despite the great advance the NTCP-hepatoma cell lines have provided, there is still some need for more robust and physiologically authentic systems that mimic more reliably the situation *in vivo*.

Interestingly, the expression of human NTCP in mouse hepatocyte cell lines confers them susceptible to HDV, but not to HBV infection [55,56,57]. An early study suggested that at least one major intracellular block to HBV infection in mouse hepatocytes is at the level of cccDNA formation [58]. A recent study found that HBV cccDNA can be formed in an immortalized mouse hepatocyte cell line and this can be correlated with the instability of HBV mature nucleocapsids in these immortalized mouse hepatocytes, suggesting that nucleocapsid uncoating may be a major intracellular determinant in the susceptibility of hepatocytes to HBV infection [59]. Another recent study indicated that it is a dependency factor, rather than a restriction factor, that is missing in mouse hepatocytes and prevents infection [60]. The identification of this critical factor, or factors, will facilitate the development of HBV infection systems based on murine hepatocytes, a requisite for establishing a mouse model for HBV infection [61] (further discussed in Section 6).

## 4. *In vitro* Systems Based on Primary Human Hepatocytes

As previously discussed, earlier attempts to establish HBV infection systems based on primary human hepatocytes have been hampered by the phenotypic instability of the cells *in vitro*, reflected by a rapid loss of their authentic hepatocyte function soon after plating accompanied by loss of permissiveness for HBV infection. This has been a major obstacle for using primary human hepatocytes to study a slow growing virus like HBV.

### 4.1. Human Fetal Hepatocytes

Several studies have attempted to use fetal human hepatocytes as a platform for HBV infection system. Ochya and colleagues have infected highly confluent cultured fetal human hepatocytes with hepatitis B virions produced by hepatoma cell line and were able to show a 12% infection efficiency with active replication that started two days after infection and accumulated during 16 days post infection [62]. The limited infection efficiency and the apparent absence of viral spreading were explained by the relative narrow window of time in which cells remained susceptible for infection, and therefore virions released into medium from infected cells possibly could not infect adjacent cells any more. Another study has similarly shown that fetal human hepatocytes could be infected with HBV infectious serum but that productive infection could remain for a limited period of time (up to 16–18 days) concomitant with the maintenance of normal hepatocytic phenotype [63]. Interestingly, the addition of DMSO appeared to enhance viral replication in this system. Another recent study has shown that co-culturing of fetal human hepatocytes with hepatic non-parenchymal cells and the subsequent addition of 2% DMSO leads to the formation of hepatocyte islands, resulting in prolonged phenotypic maintenance of those cells and susceptibility for HBV infection for up to 10 weeks [64]. However, although the above studies suggest fetal human hepatocyte as a possible platform for *in vitro* HBV studies, the limited availability of fetal hepatocytes and the large donor-to-donor variations are major limitations of this system.

### 4.2. Micro Patterned Co-Cultured Cells

Realizing that the *in vitro* maintenance of phenotypically stable primary human hepatocytes over time is a major goal not only for studying hepatotropic viruses, but also for the purpose of drug screenings as well as for metabolic and toxicity studies [9], Bhatia’s lab has integrated tissue engineering with micro technology techniques to create a miniature system of phenotypically stable primary human hepatocytes designated micro-patterned co-cultured (MPCC) system [65]. This system is based on micro-patterning of human hepatocytes in small islands of 200–400 cells each and co-culturing the cells with mouse fibroblasts, thereby providing the cells with the necessary homotypic and heterotypic cell-cell interactions to preserve their long-term viability and function. Initial studies have shown that the MPCC system preserves hepatocyte functions over weeks following their plating, as measured by their level of albumin secretion, urea synthesis, phase I and phase II enzymes activity and phase III transporter activity. Furthermore, the MPCC system has been shown to serve as a platform for drug toxicity and drug interaction studies. Last but not least, cryopreserved and not solely fresh hepatocytes could be micro-patterned and maintain their functionality over time, making the system much more practical to use.

The MPCCs has been shown to express all the required factors for hepatitis C virus (HCV) entry and to support HCV infection for several weeks [66]. This system has also been successfully used to support the hepatic stage of both, plasmodium falciparum and plasmodium vivax, and was validated as a platform for medium throughput anti-malarial drug screening [67].

More recently, MPCCs have been shown to support HBV infection, as well [68]. Following inoculation of co-cultured micro patterned cryopreserved human hepatocytes, derived from different donors, with HBV infected plasma, cells were first screened for their permissiveness for HBV infection. Quantification of cccDNA and HBV surface antigen (HBsAg) as surrogate markers for productive infection revealed a wide variability between donors in terms of HBV permissiveness. This variability could not be explained by hepatocyte phenotypic differences, since measuring albumin secretion as well as urea production and CYP3A4 activity did not differ significantly between donors. Importantly, to achieve productive infection, the inhibition of JAK-STAT pathway by Janus kinase (JAK) or TANK-binding kinase 1 (TBK1) inhibitors was required, although in several of the screened hepatocyte donors, even JAK-STAT inhibition could not rescue HBV infection. Interestingly, the HBV receptor NTCP has been expressed much more robustly on the plasma membrane of human hepatocytes seeded in the micro patterned format as compared to its expression on human hepatocytes co-cultured with mouse fibroblasts but seeded in a random manner. This suggests that micro patterning of human hepatocytes and the resulting differentiated phenotype may secure the proper expression of host factors essential for productive infection. However, some major limitations of the system are worth mentioning; first, no measurable spread of infection was noted and as judged by immunostaining for HBV core protein, infection efficiency was at the range of 30%. Second, although measurable amount of cccDNA was detected from around day 10 post infection and seemed to be rising, other measures of active gene expression and replication, such as pre-genomic RNA level, HBeAg as well HBsAg levels peaked at around day 16 post infection and declined rapidly thereafter. Third, medium collected from infected cells was not able to re-infect naïve cells, suggesting that viral production by the system was not robust enough to produce substantial infectious viral inoculum. However, despite these limitations, the system provided some important information regarding the activation of the innate immune response following HBV infection. Specifically, in addition to a detectable amount of both interferon (IFN)α and IFNβ, several anti-viral interferon-stimulated genes (ISGs) products, such as viperin, cGAS, and ISG15 among others have been induced in a temporal manner following HBV infection. This implies that, despite the general belief supported by few *in vivo* observations [69] of HBV being a “stealth virus” [70,71], this might not hold true at least in the context of the MPCC system. Interestingly, this observation is in line with other studies mainly performed in HepaRG cell line, suggesting that HBV infection is implicated in activation of the innate immune response [72,73]. However, further studies should better address this issue by focusing on inherent differences between various infection systems and their impact on the ability of HBV to induce the innate immune response. In addition, it will be interesting to test HBV infection of the MPCC system in the context of a 3D, rather than 2D, culture since liver architecture and the position of the hepatocytes relative to their neighboring parenchymal and non-parenchymal cells may play an important role in their permissiveness to HBV [74].

## 5. *In vitro* Systems Based on Induced Pluripotent Stem (iPS) Cell-Derived Human Hepatocytes

Induced pluripotent stem cells were first introduced by Yamanaka and colleagues, who forced the expression of a set of transcription factors in adult-derived cells [75,76]. The resulting pluripotent cells can remain genetically stable and self-renew in culture with the potential to be differentiated into cell lineages of all three germ layers including hepatocyte-like cells (HLCs) [77,78,79]. Notably, HLCs are typically similar to fetal hepatocytes and do not represent the full phenotypic spectrum of primary adult human hepatocytes [80]. Despite this caveat, iPS-derived HLCs have been shown to support the whole life cycle of HCV [81] and the hepatic stage of plasmodium infection, the causative agent of malaria [82]. Recently, HLCs have been also shown to support HBV infection [79]. Specifically, iPS cells were cultured and differentiated to HLCs over a 20-day differentiation process according to a well-defined protocol. A time-course experiment coupled to the differentiation process of those cells demonstrated that both a full activation of the transcription machinery and a robust expression of NTCP on the cells’ surface are essential to achieve a productive infection, reflected by cccDNA production and HBsAg secretion. Interestingly, the shift point for the cells to become HBV permissive was at around days 18–20 of differentiation, which is the time of phenotypic switch from hepatoblast-like to fetal hepatocyte like cells. Given the recent discovery of small molecules that can further differentiate HLCs into more mature phenotype that resemble adult human hepatocytes [83], it will be interesting to test weather HBV infection would be more robust under those conditions. Of note and similar to the case of MPCCs, HLCs susceptibility to infection was largely dependent on silencing the type I interferon response by using a JAK inhibitor prior to and following infection. In addition, HBV infection of HLCs resulted in the induction of a set of anti-viral ISGs in a similar pattern observed with HBV infected MPCCs.

The establishment of HBV infection system based on HLCs can serve as a platform to dissect important host factors essential for HBV infection and replication. This can be done by comparing the gene expression profile of cells just prior to and following the tipping point of HBV permissiveness followed by the establishment HLCs derived from iPS cell lines knocked-down or knocked-out for specific candidate genes. In accordance with this, the HBV receptor NTCP, known as one of the central factors induced during the late stages of HLCs differentiation provides at least a partial explanation for the late stage of differentiation in which cells become permissive for HBV infection. Recently, the p.Ser267Phe NTCP variant has been shown to confer resistance to HBV infection following genetic and epidemiologic analyses of Han Chinese cohort [84]. The production of iPS cells from those patients’ fibroblasts and their differentiation to HLCs could serve as an elegant platform to definitely prove the resistance of this variant to HBV infection *in vitro*.

## 6. Chimeric Mice Models for HBV Infection Based on Human Hepatocytes

In contrast to human hepatocytes, murine hepatocytes are not permissive for HBV infection even upon over-expression of the human homologue of NTCP and, therefore, the creation of a small animal model for HBV infection is a challenge. Long-standing *in vivo* models such as the HBV transgenic mice [85] are severely limited by their inability to tackle basic issues in HBV biology, such as viral entry as well as cccDNA formation and maintenance.

The technical and conceptual progress made with the isolation and maintenance of primary human hepatocytes paved the way towards using these cells for creating chimeric mouse models that can recapitulate the whole life cycle of HBV, a much needed tool for studying HBV *in vivo* (reviewed in [86,87]). The basic idea is to implant freshly-isolated primary human hepatocytes that can be stably integrated in the animal’s liver parenchyma. For this, one should use immune-compromised animals to avoid immunological response against the transplanted xenogenic hepatocytes and at the same time to initiate limited liver damage to create a proper niche for the engraftment and propagation of the transplanted hepatocytes. The two best characterized models are the Alb-urokinase type plasminogen activator (uPA) transgenic mouse [88,89] in which sub-acute liver failure is induced by the uPA transgene and the knockout fumarylacetoacetate hydrolase (FAH) mouse model [90,91,92] in which hypertyrosinemia and liver failure ensue unless the animals are protected by consuming the NTCB drug. Following their intra-splenic injection, the successful engraftment of human hepatocytes in the animals’ livers usually takes several weeks, during which time measurement of serum human albumin levels can be used as an indicator for the magnitude of engraftment [93].

Both the uPA and the FAH deficient mouse models have been shown to support HBV infection. Following inoculation, the virus gradually spreads in the engrafted human hepatocytes to ultimately infect the vast majority of engrafted cells [88,91,92,94]. The ability to use cryopreserved hepatocytes for liver engraftment not only made the system much more flexible and technically feasible for routine use, but also opened the door for studying viral biology and the effect of anti-viral drugs in the context of different genetic backgrounds. Furthermore, the human chimeric mouse models make it possible to use natural viruses derived from various sources, as well as recombinant mutated viruses for infection experiments to study basic questions in HBV biology and in virus-host interactions. For example, a study performed in uPA-SCID mice has shown that following HBV infection, binding of the viral pre-S1 motif to the bile-acid pump NTCP results in major alterations in the expression of genes implicated in lipid metabolism and bile-acid synthesis[95]. Those findings emphasize the intimate link between HBV infection and liver metabolism [96]. Another recent study performed using the same animal model has demonstrated that HBV and HDV co-infection results in a much more robust induction of ISGs as compared to HBV mono-infection [97]. These findings may provide a mechanism for the more severe liver damage frequently observed in co-infected patients and for the well-known phenomenon of HBV suppression by HDV among those patients.

However, *in vivo* systems for HBV infection based on engrafted human hepatocytes still have their limitations; the experiments are expansive due to the high costs of both human hepatocytes and the animals, the engraftment process is long and cumbersome, and the animals are deficient in most of the components of their immune system, precluding studies addressing host-adaptive immune system interactions. Reconstitution of a functional immune system by taking both hepatocytes and immune cells from the same donor to avoid immunological response against the xenograft [98] is one example for current efforts made to create a humanized immune-competent mouse model to study hepatotropic viruses.

## 7. Conclusions and Future Perspectives

Although emerging *in vivo* infection systems for HBV hold much promise, there is still a crucial need for *in vitro* systems mimicking HBV infection to address key questions in HBV biology. However, in a sharp contrast to the situation *in vivo*, a robust and long-lived HBV infection is extremely difficult to achieve *in vitro* in almost any cell culture system developed so far (Summarized in Table 1). The reason for this discrepancy is not clear and is a subject for speculations and hypotheses, but recent technological as well as conceptual progress has advanced the development of more robust infection systems. The development of hepatoma cell-based cultures over expressing the HBV receptor, NTCP, seems to disrupt the long-standing barrier in infecting those easy to handle cell lines. Novel co-culturing techniques with immune cells hold promise for applying this system for studies regarding HBV immunobiology, as well. Concomitantly, there is increasing effort to improve our ability to maintain primary human hepatocytes phenotypically stable for long periods of time or, alternatively, to produce hepatocyte like cells using the powerful iPS cells technology. Those systems hold promise to serve as a platform for HBV infection on a more physiologically authentic background. It is conceivable that many conclusions regarding HBV immunobiology and its interactions with the host, previously derived from artificial over-expression systems, will not prove to hold true following experiments involving more reliable *in vitro* infection systems. Thus, with the advent of more physiological and robust HBV infection systems, one can expect for renewed discoveries side by side with the fall of old concepts regarding this fascinating virus.

**Table 1:** A summary of the various *in vitro* systems for studying HBV biology.

Cell Culture Systems	Advantages	Disadvantages	Comments
***In vitro* systems based on primary non-human hepatocytes**			
Primary duck hepatocytes	Recapitulating the whole hepadnavirus life cycle.	Conclusions derived from this system (cccDNA biology, viral entry) are not necessarily valid for HBV.	Infected with DHBV.
	Increased amplification of cccDNA (ideal for studying cccDNA biology).		DHBV differs from HBV in several properties (See text for details).
Primary woodchuck hepatocytes	Recapitulating the whole hepadnavirus life cycle.	The system is hard to handle and maintain, few studies using this *in vitro* system.	Infected with WHV.
		Expensive.	The system is very useful for *in vivo* studies.
Primary tree shrews (*Tupaya Belangeri*) hepatocytes	The only species susceptible for HBV infection besides humans and chimpanzees.	Expensive	Used for *in vitro* as well as *in vivo* infection experiments.
***In vitro* systems based on hepatoma cell lines**			
Immortalized hepatoma cell lines	Convenient for work.	Not permissive for HBV infection.	Examples: HepG2, Huh7 cell lines.
	Minimal variability.	Cancerous cell lines. Virus-host interaction studies often non-reliable.	
	Relatively cheap.		
HepG2.2.15	Stable and continuous HBV gene expression and replication.	Incomplete viral life cycle (cells not permissive for infection).	Hepatoma cells with a cloned head to tail HBV dimer, extensively used for anti-viral drug testing and as a platform for *in vitro* experiments.
	A potential source for tissue culture derived virions.	Virions are produced from the integrated DNA (unlike situation *in vivo*).	
		Minimal amount of cccDNA.	
HepAD38	Tightly controlled system to study HBV.	Incomplete viral life cycle (cells not permissive for infection).	Hepatoma cells stably expressing HBV from a Tet-on/Tet-off system.
	Robust production of virions and enhanced cccDNA accumulation in the cells.		
	A potential source for tissue culture derived virions.		
HepDE19	A platform for a large-scale cccDNA-targeting drug screening (HBeAg as a surrogate marker for cccDNA abundance).	Incomplete viral life cycle (cells not permissive for infection)	HepG2 cells stably transfected with a Tet-inducible mutated HBV genome. The HBeAg is expressed from the episomal DNA (cccDNA) but not from the integrated genome.
HepaRG	Recapitulating the whole life cycle of HBV in the context of authentic infection.	The use of PEG is required to achieve infection, relatively low infection efficiency.	This cell line presents morphological and functional features similar to primary hepatocytes.
	An experimental platform for studies addressing key questions in HBV biology.	Stringent conditions needed to maintain those cells’ state of differentiation.	Susceptible to HBV infection upon supplementation of corticoids and DMSO to maintain the cells’ differentiation state.
NTCP-expressing hepatoma cell lines	Recapitulate the whole life cycle of HBV in the context of authentic infection.	The multiplicity of infection (MOI) needed to achieve infection is extremely high.	HepG2 and Huh7-based cell lines.
	Flexibility and easy handling.	In most instances PEG is needed to enhance infection.	Upon co-culturing with HBV-specific CD8 cells (trans-well system) the system can be used for immunobiology studies.
		No substantial viral spreading following infection, infection is short-lived small amount of cccDNA detected.	
		Hepatoma cells are physiologically impaired in many intracellular pathways and functions, limiting their use as a platform for studying virus-host interactions.	
***In vitro systems* based on primary human hepatocytes**			
Primary human hepatocytes	The gold standard host cell for HBV infection experiments.	Phenotypically unstable *in vitro*.	
		Rapidly lose permissiveness for HBV infection.	
		Large variability among hepatocyte donors	
		Short durability of infection.	
Fetal human hepatocytes	Phenotypically close (but not equal) to primary adult human hepatocytes.	Limited infection efficiency and apparent absence of viral spreading.	The addition of DMSO may enhance viral replication.
		Large donor-top-donor variations.	Co-culturing with hepatic non-parenchymal cells and subsequent addition of 2% DMSO leads to the formation of hepatocyte islands with prolonged phenotypic maintenance
		Limited availability.	
Micro-patterned co-cultured (MPCC) system	Preserves hepatocyte functions and viability over weeks following plating.	Wide variability between donors in terms of HBV permissiveness.	The system is based on micro-patterning of human hepatocytes in small islands of 200–400 cells each and co-culturing the cells with mouse fibroblasts.
	May serve as a platform for drug toxicity and drug interaction studies.	Infection efficiency is low (30%), no substantial spreading of infection.	
	Fresh as well as cryopreserved hepatocytes could be micro-patterned.	The inhibition of the innate immune response is required to achieve infection.	
Hepatocyte-like cells (HLCs)	Supports HBV productive infection.	Does not represent the full phenotypic spectrum of primary adult human hepatocytes (similar to fetal hepatocytes).	The shift point for the iPS cells to become HBV permissive is at around days 18–20 of differentiation, which is the time of phenotypic switch from hepatoblast-like to fetal hepatocyte like cells.
	Isogenic background.	Needs high degree of expertise. Complicated protocols involved.	
	May serve as a platform to dissect host factors essential for HBV infection and replication.	The inhibition of the innate immune response is required to achieve infection	
**Delivery vector systems**			
A recombinant HBV baculovirus system	Enables the delivery of a functional HBV genome into hepatoma cells resulting in productive HBV replication, formation of infectious viruses and establishment of a detectable intracellular cccDNA pool.	Bypassing the natural entry stage of HBV.	The vector is produced in insect cells.
		Part of the host response to HBV infection might be masked by a non-specific response to the viral vector.	
Adenovirus vector carrying the HBV genome (Ad-HBV)	Infect a wide range of hepatocytes irrespective of species barrier resulting in cccDNA formation and robust HBV replication.		

HBV: Hepatitis B virus; cccDNA: covalently closed circular DNA; DHBV: Duck HBV; WHV: Woodchuck hepatitis virus; iPS: Induced pluripotent stem; HBeAg: HBV e-Antigen; PEG: Polyethylene glycol; DMSO: Dimethyl sulfoxide

## References

[1] Ganem D., Prince A.M. (2004). Hepatitis B virus infection—Natural history and clinical consequences. N. Engl. J. Med..

[2] Ni Y., Lempp F.A., Mehrle S., Nkongolo S., Kaufman C., Fälth M., Stindt J., Königer C., Nassal M., Kubitz R. (2014). Hepatitis B and d viruses exploit sodium taurocholate co-transporting polypeptide for species-specific entry into hepatocytes. Gastroenterology.

[3] Yan H., Zhong G., Xu G., He W., Jing Z., Gao Z., Huang Y., Qi Y., Peng B., Wang H. (2012). Sodium taurocholate cotransporting polypeptide is a functional receptor for human hepatitis B and d virus. eLife.

[4] Ganem D., Varmus H.E. (1987). The molecular biology of the hepatitis B viruses. Annu Rev Biochem.

[5] Nassal M. (2015). Hbv cccdna: Viral persistence reservoir and key obstacle for a cure of chronic hepatitis B. Gut.

[6] Liang T.J., Block T.M., McMahon B.J., Ghany M.G., Urban S., Guo J.-T., Locarnini S., Zoulim F., Chang K.-M., Lok A.S. (2015). Present and future therapies of hepatitis B: From discovery to cure. Hepatology.

[7] Fields B.N., Knipe D.M., Howley P.M. (2013). Fields virology.

[8] Guillouzo A. (1998). Liver cell models in *in vitro* toxicology. Environ. Health Perspect..

[9] Hewitt N.J., Gómez Lechón M.J., Houston J.B., Hallifax D., Brown H.S., Maurel P., Kenna J.G., Gustavsson L., Lohmann C., Skonberg C. (2007). Primary hepatocytes: Current understanding of the regulation of metabolic enzymes and transporter proteins, and pharmaceutical practice for the use of hepatocytes in metabolism, enzyme induction, transporter, clearance, and hepatotoxicity studies. Drug Metab. Rev..

[10] Galle P.R., Hagelstein J., Kommerell B., Volkmann M., Schranz P., Zentgraf H. (1994). *In vitro* experimental infection of primary human hepatocytes with hepatitis B virus. Gastroenterology.

[11] Gripon P., Diot C., Thézé N., Fourel I., Loreal O., Brechot C., Guguen-Guillouzo C. (1988). Hepatitis B virus infection of adult human hepatocytes cultured in the presence of dimethyl sulfoxide. J. Virol..

[12] Tennant B.C., Gerin J.L. (2001). The woodchuck model of hepatitis B virus infection. ILAR J..

[13] Aldrich C.E., Coates L., Wu T.-T., Newbold J., Tennant B.C., Summers J., Seeger C., Mason W.S. (1989). *In vitro* infection of woodchuck hepatocytes with woodchuck hepatitis virus and ground squirrel hepatitis virus. Virology.

[14] Moraleda G., Saputelli J., Aldrich C.E., Averett D., Condreay L., Mason W.S. (1997). Lack of effect of antiviral therapy in nondividing hepatocyte cultures on the closed circular DNA of woodchuck hepatitis virus. J. Virol..

[15] Korba B.E., Cote P., Hornbuckle W., Tennant B.C., Gerin J.L. (2000). Treatment of chronic woodchuck hepatitis virus infection in the eastern woodchuck (marmota monax) with nucleoside analogues is predictive of therapy for chronic hepatitis B virus infection in humans. Hepatology.

[16] Hsu T.-y., Möröy T., Etiemble J., Louise A., Trépo C., Tiollais P., Buendia M.-A. (1988). Activation of c-myc by woodchuck hepatitis virus insertion in hepatocellular carcinoma. Cell.

[17] Korba B.E., Wells F.V., Baldwin B., Cote P.J., Tennant B.C., Popper H., Gerin J.L. (1989). Hepatocellular carcinoma in woodchuck hepatitis virus-infected woodchucks: Presence of viral DNA in tumor tissue from chronic carriers and animals serologically recovered from acute infections. Hepatology.

[18] Liu J., Zhang E., Ma Z., Wu W., Kosinska A., Zhang X., Möller I., Seiz P., Glebe D., Wang B. (2014). Enhancing virus-specific immunity *in vivo* by combining therapeutic vaccination and pd-l1 blockade in chronic hepadnaviral infection. PLoS Pathog..

[19] Menne S., Maschke J., Tolle T.K., Lu M., Roggendorf M. (1997). Characterization of t-cell response to woodchuck hepatitis virus core protein and protection of woodchucks from infection by immunization with peptides containing a t-cell epitope. J. Virol..

[20] Tuttleman J.S., Pourcel C., Summers J. (1986). Formation of the pool of covalently closed circular viral DNA in hepadnavirus-infected cells. Cell.

[21] Tuttleman J.S., Pugh J.C., Summers J.W. (1986). *In vitro* experimental infection of primary duck hepatocyte cultures with duck hepatitis B virus. J. Virol..

[22] Sprengel R., Kuhn C., Will H., Schaller H. (1985). Comparative sequence analysis of duck and human hepatitis B virus genomes. J. Med. Virol..

[23] Hantz O., Parent R., Durantel D., Gripon P., Guguen-Guillouzo C., Zoulim F. (2009). Persistence of the hepatitis B virus covalently closed circular DNA in heparg human hepatocyte-like cells. J. Gener. Virol..

[24] Breiner K.M., Urban S., Schaller H. (1998). Carboxypeptidase d (gp180), a golgi-resident protein, functions in the attachment and entry of avian hepatitis B viruses. J. Virol..

[25] Walter E., Keist R., Niederöst B., Pult I., Blum H.E. (1996). Hepatitis B virus infection of tupaia hepatocytes *in vitro* and *in vivo*. Hepatology.

[26] Acs G., Sells M.A., Purcell R.H., Price P., Engle R., Shapiro M., Popper H. (1987). Hepatitis B virus produced by transfected hep g2 cells causes hepatitis in chimpanzees. Proc. Natl. Acad. Sci. USA.

[27] Sells M.A., Chen M.-L., Acs G. (1987). Production of hepatitis B virus particles in hep g2 cells transfected with cloned hepatitis B virus DNA. Proc. Natl. Acad. Sci. USA.

[28] Ding X.R., Yang J., Sun D.C., Lou S.K., Wang S.Q. (2007). Whole genome expression profiling of hepatitis B virus-transfected cell line reveals the potential targets of anti-hbv drugs. Pharmacogenomics J..

[29] Li G.-Q., Xu W.-Z., Wang J.-X., Deng W.-W., Li D., Gu H.-X. (2007). Combination of small interfering rna and lamivudine on inhibition of human b virus replication in hepg2.2.15 cells. World J. Gastroenterology.

[30] Tsurimoto T., Fujiyama A., Matsubara K. (1987). Stable expression and replication of hepatitis B virus genome in an integrated state in a human hepatoma cell line transfected with the cloned viral DNA. Proc. Natl. Acad. Sci..

[31] Ladner S.K., Otto M.J., Barker C.S., Zaifert K., Wang G.H., Guo J.T., Seeger C., King R.W. (1997). Inducible expression of human hepatitis B virus (hbv) in stably transfected hepatoblastoma cells: A novel system for screening potential inhibitors of hbv replication. Antimicrob. Agents Chemother..

[32] Sun D., Nassal M. (2006). Stable hepg2- and huh7-based human hepatoma cell lines for efficient regulated expression of infectious hepatitis B virus. J. Hepatol..

[33] Cai D., Mills C., Yu W., Yan R., Aldrich C.E., Saputelli J.R., Mason W.S., Xu X., Guo J.T., Block T.M. (2012). Identification of disubstituted sulfonamide compounds as specific inhibitors of hepatitis B virus covalently closed circular DNA formation. Antimicrob. Agents Chemother..

[34] Guo H., Jiang D., Zhou T., Cuconati A., Block T.M., Guo J.-T. (2007). Characterization of the intracellular deproteinized relaxed circular DNA of hepatitis B virus: An intermediate of covalently closed circular DNA formation. J. Virol..

[35] Delaney W.E., Isom H.C. (1998). Hepatitis B virus replication in human hepg2 cells mediated by hepatitis B virus recombinant baculovirus. Hepatology.

[36] Lucifora J., Durantel D., Belloni L., Barraud L., Villet S., Vincent I.E., Margeridon-Thermet S., Hantz O., Kay A., Levrero M. (2008). Initiation of hepatitis B virus genome replication and production of infectious virus following delivery in hepg2 cells by novel recombinant baculovirus vector. J. Gener. Virol..

[37] Isom H., Abdelhamed A., Bilello J., Miller T., Hamatake R., Lau J.N. (2004). Baculovirus-mediated gene transfer for the study of hepatitis B virus. Hepatitis B and d protocols.

[38] Delaney W.E., Miller T.G., Isom H.C. (1999). Use of the hepatitis B virus recombinant baculovirus-hepg2 system to study the effects of (−)-β-2′,3′-dideoxy-3′-thiacytidine on replication of hepatitis B virus and accumulation of covalently closed circular DNA. Antimicrob. Agents Chemother..

[39] Chen R.Y.M., Edwards R., Shaw T., Colledge D., Delaney W.E., Isom H., Bowden S., Desmond P., Locarnini S.A. (2003). Effect of the g1896a precore mutation on drug sensitivity and replication yield of lamivudine-resistant hbv *in vitro*. Hepatology.

[40] Wilson J.M. (1996). Adenoviruses as gene-delivery vehicles. N. Engl. J. Med..

[41] Sprinzl M.F., Oberwinkler H., Schaller H., Protzer U. (2001). Transfer of hepatitis B virus genome by adenovirus vectors into cultured cells and mice: Crossing the species barrier. J. Virol..

[42] Ren S., Nassal M. (2001). Hepatitis B virus (hbv) virion and covalently closed circular DNA formation in primary tupaia hepatocytes and human hepatoma cell lines upon hbv genome transduction with replication-defective adenovirus vectors. J. Virol..

[43] Cohen D., Adamovich Y., Reuven N., Shaul Y. (2010). Hepatitis B virus activates deoxynucleotide synthesis in nondividing hepatocytes by targeting the r2 gene. Hepatology.

[44] Lucifora J., Durantel D., Testoni B., Hantz O., Levrero M., Zoulim F. (2009). Control of hepatitis B virus replication by innate response of heparg cells. Hepatology.

[45] Paran N., Geiger B., Shaul Y. (2001). Hbv infection of cell culture: Evidence for multivalent and cooperative attachment. The EMBO J..

[46] Gripon P., Rumin S., Urban S., Le Seyec J., Glaise D., Cannie I., Guyomard C., Lucas J., Trepo C., Guguen-Guillouzo C. (2002). Infection of a human hepatoma cell line by hepatitis B virus. Proc. Natl. Acad. Sci. USA.

[47] Liu Y., Li J., Chen J., Li Y., Wang W., Du X., Song W., Zhang W., Lin L., Yuan Z. (2015). Hepatitis B virus polymerase disrupts k63-linked ubiquitination of sting to block innate cytosolic DNA-sensing pathways. J Virol..

[48] Luangsay S., Gruffaz M., Isorce N., Testoni B., Michelet M., Faure-Dupuy S., Ait-Goughoulte M., Romain P., Rivoire M., Javanbakht H. (2015). Early inhibition of hepatocyte innate responses by hepatitis B virus. J. Hepatol..

[49] Lucifora J., Xia Y., Reisinger F., Zhang K., Stadler D., Cheng X., Sprinzl M.F., Koppensteiner H., Makowska Z., Volz T. (2014). Specific and nonhepatotoxic degradation of nuclear hepatitis B virus cccdna. Science.

[50] Bouezzedine F., Fardel O., Gripon P. (2015). Interleukin 6 inhibits hbv entry through ntcp down regulation. Virology.

[51] Gripon P., Cannie I., Urban S. (2005). Efficient inhibition of hepatitis B virus infection by acylated peptides derived from the large viral surface protein. J. Virol..

[52] Seeger C., Mason W.S. (2013). Sodium-dependent taurocholic cotransporting polypeptide: A candidate receptor for human hepatitis B virus. Gut.

[53] Hoh A., Heeg M., Ni Y., Schuch A., Binder B., Hennecke N., Blum H.E., Nassal M., Protzer U., Hofmann M. (2015). Hepatitis B virus-infected hepg2hntcp cells serve as a novel immunological tool to analyze the antiviral efficacy of cd8+ t cells *in vitro*. J. Virol..

[54] Yan R., Zhang Y., Cai D., Liu Y., Cuconati A., Guo H. (2015). Spinoculation enhances hbv infection in ntcp-reconstituted hepatocytes. PLoS ONE.

[55] Li H., Zhuang Q., Wang Y., Zhang T., Zhao J., Zhang Y., Zhang J., Lin Y., Yuan Q., Xia N. (2014). Hbv life cycle is restricted in mouse hepatocytes expressing human ntcp. Cell Mol. Immunol..

[56] Yan H., Peng B., He W., Zhong G., Qi Y., Ren B., Gao Z., Jing Z., Song M., Xu G. (2013). Molecular determinants of hepatitis B and d virus entry restriction in mouse sodium taurocholate cotransporting polypeptide. J. Virol..

[57] He W., Ren B., Mao F., Jing Z., Li Y., Liu Y., Peng B., Yan H., Qi Y., Sun Y. (2015). Hepatitis D virus infection of mice expressing human sodium taurocholate co-transporting polypeptide. PLoS Pathog..

[58] Guidotti L.G., Matzke B., Schaller H., Chisari F.V. (1995). High-level hepatitis B virus replication in transgenic mice. J. Virol..

[59] Cui X., Clark D.N., Liu K., Xu X.-D., Guo J.-T., Hu J. (2016). Viral DNA-dependent induction of innate immune response to hepatitis B virus in immortalized mouse hepatocytes. J. Virol..

[60] Lempp F.A., Mutz P., Lipps C., Wirth D., Bartenschlager R., Urban S. (2015). Evidence that hepatitis B virus replication in mouse cells is limited by the lack of a host cell dependency factor. J. Hepatol..

[61] Winer B.Y., Ploss A. (2015). Determinants of hepatitis B and delta virus host tropism. Curr. Opin. Virol..

[62] Ochiya T., Tsurimoto T., Ueda K., Okubo K., Shiozawa M., Matsubara K. (1989). An *in vitro* system for infection with hepatitis B virus that uses primary human fetal hepatocytes. Proc. Natl. Acad. Sci. USA.

[63] Lin M., Chen Q., Yang L.-Y., Li W.-Y., Cao X.-B., Wu J.-R., Peng Y.-P., Chen M.-R. (2007). Hepatitis B virus infection and replication in primarily cultured human fetal hepatocytes. World J. Gastroenterol..

[64] Zhou M., Zhao F., Li J., Cheng Z., Tian X., Zhi X., Huang Y., Hu K. (2014). Long-term maintenance of human fetal hepatocytes and prolonged susceptibility to hbv infection by co-culture with non-parenchymal cells. J. Virol. Methods.

[65] Khetani S.R., Bhatia S.N. (2008). Microscale culture of human liver cells for drug development. Nat. Biotech..

[66] Ploss A., Khetani S.R., Jones C.T., Syder A.J., Trehan K., Gaysinskaya V.A., Mu K., Ritola K., Rice C.M., Bhatia S.N. (2010). Persistent hepatitis C virus infection in microscale primary human hepatocyte cultures. Proc. Natl. Acad. Sci. USA.

[67] March S., Ng S., Velmurugan S., Galstian A., Shan J., Logan D., Carpenter A., Thomas D., Lee Sim B.K., Mota M.M. (2013). A microscale human liver platform that supports the hepatic stages of plasmodium falciparum and vivax. Cell Host Microbe.

[68] Shlomai A., Schwartz R.E., Ramanan V., Bhatta A., de Jong Y.P., Bhatia S.N., Rice C.M. (2014). Modeling host interactions with hepatitis B virus using primary and induced pluripotent stem cell-derived hepatocellular systems. Proc. Natl. Acad. Sci. USA.

[69] Wieland S., Thimme R., Purcell R.H., Chisari F.V. (2004). Genomic analysis of the host response to hepatitis B virus infection. Proc. Natl. Acad. Sci. USA.

[70] Bertoletti A., Ferrari C. (2012). Innate and adaptive immune responses in chronic hepatitis B virus infections: Towards restoration of immune control of viral infection. Gut.

[71] Ferrari C. (2014). Hbv and the immune response. Liver Int..

[72] Durantel D., Zoulim F. (2009). Innate response to hepatitis B virus infection: Observations challenging the concept of a stealth virus. Hepatology.

[73] Lucifora J., Durantel D., Testoni B., Hantz O., Levrero M., Zoulim F. (2010). Control of hepatitis B virus replication by innate response of heparg cells. Hepatology.

[74] Ramanan V., Scull M.A., Sheahan T.P., Rice C.M., Bhatia S.N. (2014). New methods in tissue engineering: Improved models for viral infection. Annu. Rev. Virol..

[75] Takahashi K., Tanabe K., Ohnuki M., Narita M., Ichisaka T., Tomoda K., Yamanaka S. (2007). Induction of pluripotent stem cells from adult human fibroblasts by defined factors. Cell.

[76] Takahashi K., Yamanaka S. (2006). Induction of pluripotent stem cells from mouse embryonic and adult fibroblast cultures by defined factors. Cell.

[77] Si-Tayeb K., Noto F.K., Nagaoka M., Li J., Battle M.A., Duris C., North P.E., Dalton S., Duncan S.A. (2010). Highly efficient generation of human hepatocyte–like cells from induced pluripotent stem cells. Hepatology.

[78] Touboul T., Hannan N.R.F., Corbineau S., Martinez A., Martinet C., Branchereau S., Mainot S., Strick-Marchand H., Pedersen R., Di Santo J. (2010). Generation of functional hepatocytes from human embryonic stem cells under chemically defined conditions that recapitulate liver development. Hepatology.

[79] Schwartz R.E., Fleming H.E., Bhatia S.N. (2014). Pluripotent stem cell-derived hepatocyte-like cells. Biotech. Adv..

[80] Yu Y., Liu H., Ikeda Y., Amiot B.P., Rinaldo P., Duncan S.A., Nyberg S.L. (2012). Hepatocyte-like cells differentiated from human induced pluripotent stem cells: Relevance to cellular therapies. Stem Cell Res..

[81] Schwartz R.E., Trehan K., Andrus L., Sheahan T.P., Ploss A., Duncan S.A., Rice C.M., Bhatia S.N. (2012). Modeling hepatitis C virus infection using human induced pluripotent stem cells. Proc. Natl. Acad. Sci. USA.

[82] Ng S., Schwartz R.E., March S., Galstian A., Gural N., Shan J., Prabhu M., Mota M.M., Bhatia S.N. (2015). Human ipsc-derived hepatocyte-like cells support plasmodium liver-stage infection *in vitro*. Stem Cell Rep..

[83] Shan J., Schwartz R.E., Ross N.T., Logan D.J., Thomas D., Duncan S.A., North T.E., Goessling W., Carpenter A.E., Bhatia S.N. (2013). Identification of small molecules for human hepatocyte expansion and ips differentiation. Nat. Chem. Biol..

[84] Peng L., Zhao Q., Li Q., Li M., Li C., Xu T., Jing X., Zhu X., Wang Y., Li F. (2015). The p.Ser267phe variant in slc10a1 is associated with resistance to chronic hepatitis B. Hepatology.

[85] Chisari F.V. (1995). Hepatitis B virus transgenic mice: Insights into the virus and the disease. Hepatology.

[86] Dandri M., Lütgehetmann M. (2014). Mouse models of hepatitis B and delta virus infection. J. Immunol. Methods.

[87] Gaska J.M., Ploss A. (2015). Study of viral pathogenesis in humanized mice. Curr. Opin. Virol..

[88] Dandri M., Burda M.R., Török E., Pollok J.M., Iwanska A., Sommer G., Rogiers X., Rogler C.E., Gupta S., Will H. (2001). Repopulation of mouse liver with human hepatocytes and *in vivo* infection with hepatitis B virus. Hepatology.

[89] Sandgren E.P., Palmiter R.D., Heckel J.L., Daugherty C.C., Brinster R.L., Degen J.L. (1991). Complete hepatic regeneration after somatic deletion of an albumin-plasminogen activator transgene. Cell.

[90] Azuma H., Paulk N., Ranade A., Dorrell C., Al-Dhalimy M., Ellis E., Strom S., Kay M.A., Finegold M., Grompe M. (2007). Robust expansion of human hepatocytes in fah-/-/rag2-/-/il2rg-/- mice. Nat. Biotech..

[91] Bissig K.-D., Le T.T., Woods N.-B., Verma I.M. (2007). Repopulation of adult and neonatal mice with human hepatocytes: A chimeric animal model. Proc. Natl. Acad. Sci. USA.

[92] Bissig K.-D., Wieland S.F., Tran P., Isogawa M., Le T.T., Chisari F.V., Verma I.M. (2010). Human liver chimeric mice provide a model for hepatitis B and c virus infection and treatment. The J. Clin. Investig..

[93] Dandri M., Petersen J. (2012). Chimeric mouse model of hepatitis B virus infection. J. Hepatol..

[94] Meuleman P., Leroux-Roels G. (2008). The human liver-upa-scid mouse: A model for the evaluation of antiviral compounds against hbv and hcv. Antivir. Res..

[95] Oehler N., Volz T., Bhadra O.D., Kah J., Allweiss L., Giersch K., Bierwolf J., Riecken K., Pollok J.M., Lohse A.W. (2014). Binding of hepatitis B virus to its cellular receptor alters the expression profile of genes of bile acid metabolism. Hepatology.

[96] Shlomai A., Shaul Y. (2008). The “metabolovirus” model of hepatitis B virus suggests nutritional therapy as an effective anti-viral weapon. Med. Hypotheses.

[97] Giersch K., Allweiss L., Volz T., Helbig M., Bierwolf J., Lohse A.W., Pollok J.M., Petersen J., Dandri M., Lütgehetmann M. (2015). Hepatitis Delta co-infection in humanized mice leads to pronounced induction of innate immune responses in comparison to hbv mono-infection. J. Hepatol..

[98] Washburn M.L., Bility M.T., Zhang L., Kovalev G.I., Buntzman A., Frelinger J.A., Barry W., Ploss A., Rice C.M., Su L. (2011). A humanized mouse model to study hepatitis C virus infection, immune response, and liver disease. Gastroenterol..

